# In vivo tissue temperatures during 90 W/4 sec‐very high power‐short‐duration (vHPSD) ablation versus ablation index‐guided 50 W‐HPSD ablation: A porcine study

**DOI:** 10.1111/jce.15782

**Published:** 2022-12-26

**Authors:** Naoto Otsuka, Yasuo Okumura, Sayaka Kuorkawa, Koichi Nagashima, Yuji Wakamatsu, Satoshi Hayashida, Kimie Ohkubo, Toshiko Nakai, Hiroyuki Hao, Rie Takahashi, Yoshiki Taniguchi

**Affiliations:** ^1^ Department of Medicine, Division of Cardiology Nihon University School of Medicine Tokyo Japan; ^2^ Department of Pathology and Microbiology Nihon University School of Medicine Division of Human Pathology Tokyo Japan; ^3^ Section of Laboratory for Animal Experiments, Institute of Medical Science, Medical Research Support Center Nihon University School of Medicine Tokyo Japan

**Keywords:** atrial fibrillation, catheter ablation, esophageal injury, pulmonary vein, tissue temperature

## Abstract

**Introduction:**

Neither the actual in vivo tissue temperatures reached with 90 W/4 s‐very high‐power short‐duration (vHPSD) ablation for atrial fibrillation nor the safety and efficacy profile have been fully elucidated.

**Methods:**

We conducted a porcine study (*n* = 15) in which, after right thoracotomy, we implanted 6–8 thermocouples epicardially in the superior vena cava, right pulmonary vein, and esophagus close to the inferior vena cava. We compared tissue temperatures close to a QDOT MICRO catheter, between during 90 W/4 s‐vHPSD ablation during ablation index (AI: target 400)‐guided 50 W‐HPSD ablation, both targeting a contact force of 8–15 g.

**Results:**

Maximum tissue temperature reached during 90 W/4 s‐vHPSD ablation did not differ significantly from that during 50 W‐HPSD ablation (49.2 ± 8.4°C vs. 50.0 ± 12.1°C; *p* = .69) and correlated inversely with distance between the catheter tip and the thermocouple, regardless of the power settings (*r* = −0.52 and *r* = −0.37). Lethal temperature (≥50°C) was best predicted at a catheter tip‐to‐thermocouple distance cut‐point of 3.13 and 4.27 mm, respectively. All lesions produced by 90 W/4 s‐vHPSD or 50 W‐HPSD ablation were transmural. Although there was no difference in the esophageal injury rate (50% vs. 66%, *p* = .80), the thermal lesion was significantly shallower with 90 W/4 s‐vHPSD ablation than with 50W‐HPSD ablation (381.3 ± 127.3 vs. 820.0 ± 426.1 μm from the esophageal adventitia; *p* = .039).

**Conclusion:**

Actual tissue temperatures reached with 90 W/4 s‐vHPSD ablation appear similar to those with AI‐guided 50 W‐HPSD ablation, with the distance between the catheter tip and target tissue being shorter for the former. Although both ablation settings may create transmural lesions in thin atrial tissues, any resulting esophageal thermal lesions appear shallower with 90 W/4 s‐vHPSD ablation.

## Graphical abstract

Actual tissue temperatures, the tip‐to‐target tissue distance needed to achieve a lethal temperature, and esophageal injury with 90 W/4 s‐very high power‐short‐duration (vHPSD) ablation versus ablation index‐guided 50 W‐HPSD ablation.

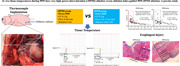

## 1 INTRODUCTION

Recent clinical studies have shown high power (≥40 W, usually 50 W) short‐duration (HPSD) ablation to be both efficacious and safe for treatment of atrial fibrillation (AF) and that the strategy decreases procedure time.^1^, ^2^ Results of several in vitro and ex vivo animal studies have suggested that application of this strategy can restrict conductive heating and increase resistive heating to allow targeted heating of the atrial wall, reducing the risk of collateral damage.^3^, ^4^, ^5^, ^6^ In a recent in vivo porcine study, we found tissue temperatures to be higher during ablation index (AI)‐guided 50 W‐HPSD ablation than during 30 W‐standrd ablation, both performed with a THERMOCOOL SMARTTOUCH SF (STSF) catheter (Biosense Webster), and we observed mild collateral phrenic nerve damage and esophageal injury even with 50 W‐HPSD ablation.^7^ Recently, the QDOT MICRO catheter (Biosense Webster) was developed and incorporated into the CARTO 3 System (Biosense Webster).^5^, ^8^ This catheter has six thermocouples within the outer metal shell of the catheter tip, and therefore, tissue temperature is reflected more accurately than it was during use of the STSF catheter, allowing for temperature‐controlled ablation. With this new catheter, one can select between two ablation modes, i.e., very HPSD (vHPSD) ablation mode (Qmode+) with 90 W power, maintaining the target temperature for only 4 s, and conventional standard ablation mode (Qmode) with up to 50 W power for temperature‐controlled ablation. In a preclinical model, 90 W/4 s‐vHPSD ablation was shown to produce wider lesions at a similar depth and to improve lesion‐to‐lesion uniformity, linear contiguity, and transmurality with a safety profile similar to that of conventional 25 W/20 s‐ablation.^5^ The subsequent first‐in‐human trial of use of the QDOT MICRO catheter for vHPSD ablation showed the clinical feasibility and safety of 90 W/4 s‐vHPSD ablation.^8^ However, with respect to 90 W/4 s‐vHPSD ablation, in vivo tissue temperatures and pathological findings, including the esophageal and phrenic nerve tissue, have not been fully documented, even in preclinical trials. Thus, we conducted an animal study in which we investigated actual tissue temperatures reached and the mechanism of heating by 90 W/4 s‐vHPSD ablation in comparison to those accompanying 50 W‐HPSD ablation guided by an AI of 400 at the pulmonary vein (PV) orifice, superior vena cava (SVC), and esophagus close to the inferior vena cava (IVC). We also sought to identify factors involved in 90 W/4 s‐vHPSD‐associated phrenic nerve and esophageal injury.

## 2 METHODS

Data supporting the findings of this study are available from the corresponding author upon reasonable request.

### 2.1 Animal preparation

The experimental protocol was approved by the Institutional Animal Care and Use Committee of Nihon University School of Medicine. Fifteen pigs were prepared for the study, as previously described.^7^, ^9^, ^10^, ^11^ After establishment of deep anesthesia, vascular access was obtained percutaneously, and continuous surface electrocardiogram and blood pressure monitoring were performed.

### 2.2 Thermocouple implantation

**Figure 1:**
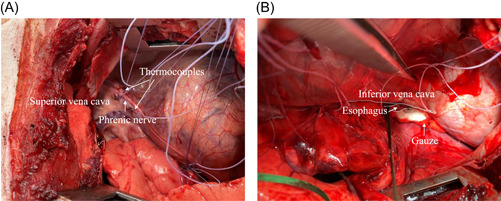
Photographs showing the superior vena cava and esophagus thermocouple implantation sites. Two thermocouples were implanted in the superior vena cava (A). After implantation of two thermocouples in the esophagus, gauze was placed underneath the esophagus to position it next to the inferior vena cave (B).

Thermocouple implantation and recording methods were as reported previously.^7^, ^9^, ^10^, ^11^ After right‐sided thoracotomy, 6–8 Type T (copper‐constantan) thermocouples (Physitemp Instruments; LLC) (time constant: 0.1 s; diameter: 0.064 mm; accuracy: ±0.42°C) were implanted epicardially at the SVC (3.0 ± 0.0 thermocouples) along the phrenic nerve (Figure 1A), at the right PV (RPV) (2.0 ± 0.0 thermocouples), and near the esophagus close to the IVC (2.1 ± 0.6 thermocouples) (Figure 1B). A radiopaque marker was attached to the tip of each thermocouple to aid in fluoroscopic localization. In pigs, the esophagus lies away from the inferior PVs, so, after placing two or three thermocouples in the esophagus close to the IVC, we inserted gauze underneath the esophagus to position it as close as possible to the IVC (Figure 1B). Each animal's chest was then closed to carefully reapproximate all tissues. Tissue temperatures were measured every 1.0 s. The temperature measurement system was calibrated before each ablation session.

### 2.3 90 W/4 s‐vHPSD and AI‐guided 50 W‐HPSD ablation settings

Transseptal catheterization was performed with an 8.5 Fr SL0 sheath (Abbott, Inc.) under fluoroscopic and phased‐array intracardiac ultrasound guidance. Electroanatomic mapping was performed by means of the CARTO 3 System with a 7.5F, 3.5 mm‐tip contact force (CF)‐sensing ablation catheter (QDOT MICRO). This catheter has an irrigated‐tip electrode (66 tiny irrigation holes around the electrode) with three microrecording electrodes at the distal end, six thermocouples within the electrode (three at the distal end and three at the proximal end, located only 75 μm from the surface).^5^, ^8^ The QDOT MICRO catheter was advanced to the SVC, RPV orifice, and IVC, with its positions verified by multiple measurements taken on fluoroscopy and by the 3D mapping system.^7^ One or two radiofrequency (RF) energy applications were delivered as close as possible to a target thermocouple but to avoid creation of overlapping lesions that could affect pathological assessment. RF energy was delivered randomly (basically at 1:1 allocation) to include both 90 W/4 s‐vHPSD ablation (power: 90 W; irrigation flow: 8 ml/min; electrode temperature limited to 55°C; CF: 8–15 g) and AI‐guided 50 W‐HPSD ablation (power: 50 W; electrode temperature limited to 47°C; irrigation flow: 4 or 15 ml/min; CF: 8–15 g, target AI: 400). We used a target AI of 400 because that setting has been widely used as a minimum limit of the target AI for posterior LA lesions for a circumferential PVI,^12^ and we previously found that a transmural lesion formation was achieved for all lesions when using the STSF catheter.^7^

### 2.4 Tissue temperature analysis

Energy delivery times and tissue temperatures were digitally recorded for subsequent analysis. A tissue temperature of ≥50°C was considered lethal, that is, the point at which myocardial tissue necrosis commenced.^3^, ^13^ The actual distance between the QDOT MICRO catheter tip and the thermocouple (tip‐to‐thermocouple distance) was calculated from the maximum distance measured on digitally acquired rotational fluoroscopic images taken from the right lateral side to the left lateral side and accounting for the diameter of the catheter tip.^7^, ^9^, ^10^, ^11^ All measurements were obtained at the same point in the respiratory cycle. We evaluated the following: (1) association between peak tissue temperature (Tpeak) at the thermocouple and the tip‐to‐thermocouple distance; (2) maximum tissue temperature (Tmax) and time to lethal tissue temperature ≥ 50°C (t−T ≥ 50°C) when the thermocouple was located ≤ 5 mm from the catheter tip; and (3) time to Tpeak (t‐Tpeak), thermal latency from ablation time to t‐Tpeak, and time from t‐Tpeak to baseline temperature (t‐Tbase) at all thermocouples.

### 2.5 Assessment of phrenic nerve function

During ablation targeting the SVC, the phrenic nerve was paced by a 5 Fr decapolar electrode catheter (Snake®; Japan Lifeline) at 20 mA from the SVC to control movement of the diaphragm. Ablation was terminated if phrenic nerve capture was lost. Phrenic nerve injury was categorized physiologically as transient if loss of capture or a decline in diaphragm motion was restored within 5 min after the termination of energy delivery or as persistent if loss of capture was complete and diaphragm motion was not restored within 30 min after the last energy delivery.

### 2.6 Pathological examination

After ablation, all pigs were euthanized by intracardiac injection of KCl, and the thoracic organs were removed en bloc with the pericardium intact. Each ablated SVC, RPV, IVC, and the esophagus were verified by the locations of the implanted thermocouples and then grossly examined. We used Image J software (US National Institutes of Health; Bethesda) to measure the width of the endocardial lesions in gross pathology using a digital caliper with a 0.3 mm/pixel average resolution. SVC, RPV, IVC, phrenic nerve, and esophageal segments were fixed in formalin, embedded in paraffin, and serially sectioned at 4 µm for hematoxylin‐eosin and Masson trichrome staining. Whether the ablation lesions were transmural or not, and whether collateral damage was present or not were histologically assessed. The maximum depth of the esophageal injury and area of the esophageal injury were histologically measured using the Image J software.

### 2.7 Statistical analysis

The study variables are shown as mean ± standard deviation values or as the number and/or percentage of data points obtained. Differences in variables between 90 W/4 s‐vHPSD ablation and 50 W‐HPSD ablation were analyzed by two‐sample *t* test or Fisher's exact test, as appropriate. The correlation between Tmax and tip‐to‐thermocouple distance was assessed by means of logarithmic curve‐fitting. Receiver operating characteristic (ROC) curves were drawn to identify the best cut‐point distance for prediction of lethal tissue temperature (≥50°C). All statistical analyses were performed with SAS 9.1.3 software and a two‐tailed *t*‐test, and *p* < .05 was considered statistically significant.

## 3 RESULTS

### 3.1 Tissue temperature profiles

**Figure 2:**
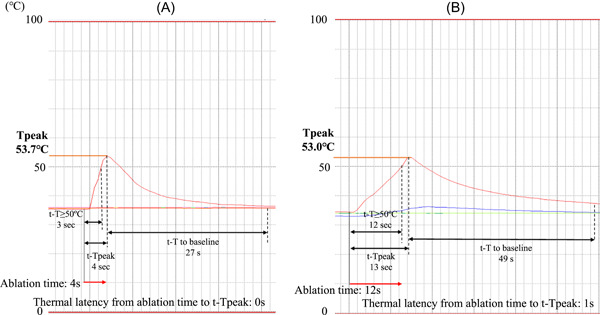
Representative time‐versus‐temperature curves for 90 W/4 s‐vHPSD ablation and 50W‐HPSD ablation. (A) With 90 W/4 s‐vHPSD ablation, tissue temperature increased rapidly (4 s) to 53.7°C when the catheter tip was positioned ≤5 mm away. (B) With 50W‐HPSD ablation guided by ablation index of 400, gradual heating resulted in a modest increase in temperature, reaching 53.0°C at 13 s at a tip‐to‐tissue distance of ≤5 mm. HSPD indicates high power short‐duration; Tpeak, peak tissue temperature; t‐T ≥ 50°C, time to lethal tissue temperature (≥50°C) when the thermocouple was located ≤ 5 mm from the catheter tip; t‐Tpeak, time to Tpeak; t‐T to baseline, time from t‐Tpeak to baseline temperature; vHPSD, very HPSD.

**Figure 3:**
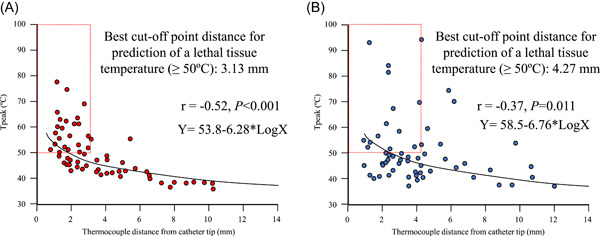
Scatterplots showing the relationship between the Tpeak and tip‐to‐thermocouple distance for 90 W/4 s‐vHPSD ablation and 50 W‐HPSD ablation. Tpeak was shown to correlate inversely with tip‐to‐thermocouple distance during 90 W/4 s‐vHSPD ablation (A) and 50 W‐HPSD ablation guided by ablation index of 400 (B). HPSD indicates high power short‐duration; Tpeak, peak tissue temperature; vHPSD, very HPSD.

Among the 15 pigs, energy was delivered 45 times either within the SVC or at the SVC‐right atrium junction, 22 times within the RPV, and 30 times within the IVC near the esophagus, yielding 60 time‐to‐tissue temperature profiles for the SVC, 24 for the RPV, and 35 for the esophagus (with 4.4 ± 1.3 profiles obtained per pig from 2.9 ± 0.4 SVC energy applications, 4.7 ± 1.1 profiles from 4.3 ± 0.8 RPV energy applications, and 3.4 ± 2.2 profiles from 2.6 ± 1.3 IVC energy applications). Of the total 119 profiles, 60 were generated from 90 W/4 s‐vHPSD ablation and 59 from AI‐guided 50W‐HPSD ablation. Representative time‐to‐tissue temperature profiles, one generated from 90 W/4 s‐vHPSD ablation and one from 50W‐HPSD ablation are shown in Figure 2A,B. The details pertaining to the tissue temperatures reached with 90 W/4 s‐vHPSD ablation and with 50 W‐HPSD ablation are summarized in Table 1. The ablation time was significantly shorter for 90 W/4 s‐vHPSD ablation than for 50 W‐HPSD ablation (4.0 ± 0 vs. 12.6 ± 2.1 s, *p* < .001). Tpeak correlated inversely with tip‐to‐thermocouple distance during each of the two ablation procedures (90 W/4 s‐vHPSD ablation: *r* = −0.52, *p* < .001; 50 W‐HPSD ablation: *r* = −0.37, *p* = .011) (Figure 3). However, Tmax measured when the catheter tip was ≤5 mm from the thermocouple did not differ significantly between 90 W/4 s‐vHPSD ablation and 50 W‐HPSD ablation (49.2 ± 8.4°C vs. 50.0 ± 12.1°C; *p* = .69) (Table 1). t−T ≥ 50°C was significantly shorter with 90 W/4 s‐vHPSD ablation than with 50 W‐HPSD ablation (4.3 ± 2.7 vs. 8.6 ± 3.2 s, respectively; *p* < .001), as was t‐Tpeak (6.8 ± 2.7 vs. 14.7 ± 3.9 s, respectively; *p* < .001). t‐Tbase was significantly shorter with 90 W/4 s‐vHPSD ablation than with 50 W‐HPSD ablation (29.9 ± 16.3 vs. 49.5 ± 23.2 s, respectively; *p* < .001). No steam pop with impedance rise occurred with either setting. Analysis of ROC curves (areas under the curve: 0.83 and 0.88 for 90 W/4 s‐vHPSD ablation and 50W‐HPSD ablation, respectively) showed a tip‐to‐thermocouple distance cut‐point of 3.13 mm (indicative of the estimated lesion depth) had the best performance for predicting achievement of a lethal temperature (≥50°C) by 90 W/4 s‐vHPSD ablation, and that of 4.27 mm performed best for predicting such achievement by 50 W‐HPSD ablation (Figure 3A,B).

**Table 1:** Total tissue temperature profiles obtained, ablation settings, and measured values, per 90 W/4 s‐vHPSD and 50 W‐HPSD (standard) ablation

	90 W/4 s‐vHPSD	50 W‐HPSD	*p* Value^a^
Total profiles (*n*)	60	59	
Total energy applications (*n*)	52	45	
Ablation parameters			
CF (g)	10.7 ± 2.1	10.2 ± 2.0	.53
Ablation time (seconds)	4.0 ± 0	12.6 ± 2.1	<.001
Impedance drop (Ω)	18.3 ± 5.1	15.5 ± 5.1	.15
Maximum electrode temperature (°C)	57.2 ± 5.1	47.7 ± 2.5	<.001
Average energy power (W)	69.6 ± 16.4	43.1 ± 5.4	<.001
Maximum power (W)	87.4 ± 5.7	50.1 ± 0.6	<.001
Tissue temperature parameters			
Tpeak (°C)	47.1 ± 9.0	49.0 ± 12.0	.32
≤5 mm (Tmax)	49.2 ± 8.4	50.0 ± 12.1	.69
>5 mm	39.9 ± 7.3	45.7 ± 6.3	.10
t‐T ≥ 50°C (s)	4.3 ± 2.7	8.6 ± 3.2	<.001
t‐Tpeak (s)	6.8 ± 2.7	14.7 ± 3.9	<.001
Thermal latency (s)	1.5 ± 1.1	1.7 ± 1.3	.65
t‐Tbase (s)	29.9 ± 16.3	49.5 ± 23.2	<.001

*Note*: Mean ± SD or median values are shown unless otherwise indicated.

Abbreviations: CF, indicates contact force; HPSD, high power short‐duration; Tmax, maximum temperature at a catheter distance of ≤5 mm; Tpeak, peak tissue temperature; thermal latency, Δ t‐Tpeak‐ ablation time; t‐T ≥ 50°C, time to lethal tissue temperature (≥50°C) at a catheter distance of ≤5 mm; t‐Tpeak, time to the peak tissue temperature; t‐Tbase, time from t‐Tpeak to baseline temperature; vHPSD, very HPSD.

^a^ By Student *t* test.

### 3.2 Relation between tissue temperatures and pathological findings

**Figure 4:**
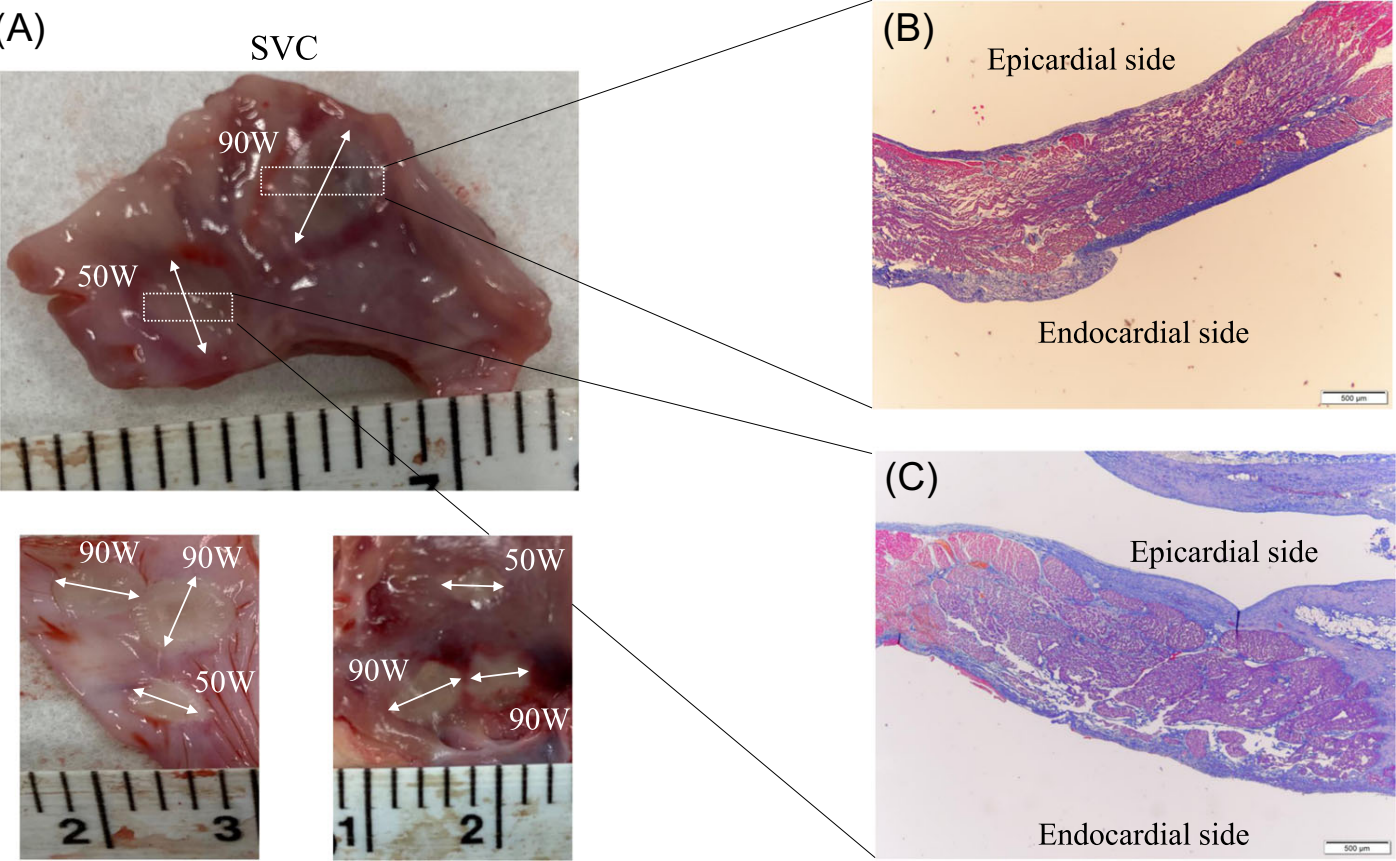
Gross pathological and histological features of the superior vena cava lesions. On gross pathology, the 90 W/4 s‐vHPSD ablation‐generated lesion was found to be wider (↔) than the 50 W‐HPSD ablation‐generated lesion (A). On histological examination (Masson's Trichrome), lesions were found to be transmural after both in 90 W/4 s‐vHPSD ablation (B) and 50 W‐HPSD ablation (C). HPSD indicates high power short‐duration; vHPSD, very HPSD.

**Figure 5:**
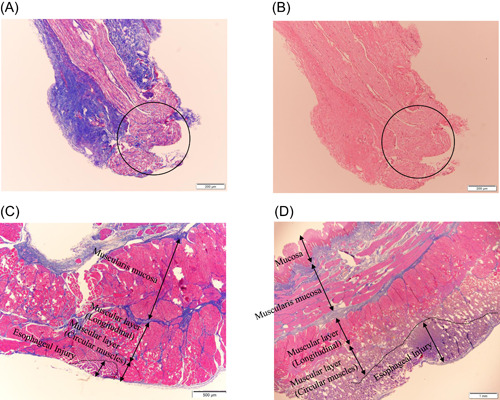
Histological findings in a case in which phrenic nerve injury occurred during 90 W/4 s‐vHPSD ablation (A and B) and in cases in which esophageal injury occurred during 90 W/4 s‐HPSD ablation (C) and 50 W‐HPSD ablation (D). Phrenic nerve segments stained with hematoxylin and eosin (A) and Masson's Trichrome (B) show thermal injury (circles), evidenced by fading or smearing of nuclear chromatin and fibrin deposition. In this case, persistent physiologic injury occurred at 50.7°C, 3 s after the start of energy delivery during 90 W/4 s‐vHPSD. In both settings, injury is seen on the adventitial surface of the esophagus and in the muscular layers, but there is no evidence of a lesion in the mucosal layers (C, D). The maximum depth between the esophageal adventitia and injury site is less (355 μm) with 90 W/4 s‐vHPSD than with 50 W‐HPSD ablation (1930 μm). The area of esophageal injury is shown by the dotted lines (C, D). In the case shown in (C), Tpeak had increased to 64.6°C, 4 s after that start of 90 W/4 s‐vHPSD ablation versus an increase to 63.6°C in the case shown in (D), 12 s after the start of 50 W‐HPSD ablation. HPSD indicates high power short‐duration; Tpeak, peak tissue temperature; vHPSD, very HPSD

Mean overall wall thickness of the SVC, RPV, and IVC was 1.37 ± 0.37 mm (range: 0.75–1.93 mm). All 97 lesions, regardless of whether they were created by 90 W/4 s‐vHPSD ablation or 50 W‐HPSD ablation, were histologically transmural, but as measured on gross pathology, endocardial lesions created by 90 W/4 s‐vHPSD ablation were wider than those created by 50 W‐HPSD ablation (6.8 ± 1.7 vs. 5.0 ± 2.0 mm, *p* = .007) (Figure 4A,B,C). With 5 (29.4%) of 17 90 W/4 s‐vHPSD energy applications at the SVC thermocouple adjacent to the phrenic nerve, persistent phrenic nerve injury occurred 3.2 ± 0.7 s after the start of energy delivery, whereas with 3 (27.3%) of 11 50 W‐HPSD energy applications (*p* = .90 vs. 90 W/4 s‐vHPSD ablation), such injury occurred at 8.5 ± 0.5 s (*p* < .001 vs. 90 W/4 s‐vHPSD ablation). However, there was no significant difference in Tpeak or tip‐to‐thermocouple distance between 90 W/4 s‐vHPSD ablation and 50 W‐HPSD ablation (Table 2). Phrenic nerve injury was observed histologically at all sites of persistent phrenic nerve injury (Figure 5A,B). Esophageal injury was similarly confirmed histologically in relation to 9 (50%) of 18 90 W/4 s‐vHPSD ablations and 8 (66%) of 12 50 W‐HPSD ablations (*p* = .80), but the maximum depth from esophageal adventitia was significantly shorter with 90 W/4 s‐vHPSD ablation than with 50 W‐HPSD ablation (381.3 ± 127.3 vs. 820.0 ± 426.1 μm; *p* = .039) (Figure 5C,D) (Table 2). There was no difference in Tpeak (50.1 ± 9.7°C vs. 52.6 ± 16.5°C; *p* = .51), but there was a modestly shorter tip‐to‐thermocouple distance (3.3 ± 1.8 vs. 4.3 ± 2.4 mm; *p* = .10) with 90 W/4 s‐vHPSD ablation than with 50 W‐HPSD ablation (Table 2).

**Table 2:** Variables assessed in relation to potential collateral damage, per 90 W/4 s‐vHPSD and 50 W‐HPSD (standard) ablation

	90 W/4 s‐vHPSD	50 W‐HPSD	*p* Value^a^
SVC profiles (*n*)	35	25	
Energy applications (*n*)	28	17	
Tpeak (°C)	46.2 ± 7.6	50.3 ± 11.9	.12
Distance^b^ (mm)	3.7 ± 2.9	3.0 ± 1.3	.30
Physiological injury (*n*)	5/17 (29.4%)	3/11 (27.3%)	.90
Histological injury (*n*)	5 (100%)	3 (100%)	
Phrenic nerve injury time (s)	3.2 ± 0.7	8.5 ± 0.5	<.001
Esophagus profiles (*n*)	19	16	
Energy applications (*n*)	18	12	
Tpeak (°C)	50.1 ± 9.7	52.6 ± 16.5	.51
Distance^b^ (mm)	3.3 ± 1.8	4.3 ± 2.4	.10
Histologic injury (*n*)	9 (50.0%)	8 (66%)	.80
Maximum distance of the injury site from esophageal adventitia (μm)	381.3 ± 127.3	820.0 ± 426.1	.039
Area of esophageal injury (mm^2^)	1.0 ± 1.3	3.1 ± 2.1	.21

*Note*: Mean ± SD values are shown unless otherwise indicated.

Abbreviations: HPSD, indicates high power short‐duration; Tpeak, peak tissue temperature; vHPSD, very HPSD.

^a^ By Student *t* test or Fisher's exact test;

^b^ Distance between the catheter tip and target tissue.

## 4 DISCUSSION

Our study yielded three major findings. First, tissue temperature with both 90 W/4 s‐vHPSD ablation and AI‐guided 50 W‐HPSD ablation correlated negatively with distance from the tip of the ablation catheter to the target tissue, but Tmax measured when the catheter tip was ≤5 mm from the thermocouple did not differ significantly between 90 W/4 s‐vHPSD ablation and 50 W‐HPSD ablation. Second, lethal tissue temperature (≥50°C) was reached more quickly with 90 W/4 s‐vHPSD ablation than with 50 W‐HPSD ablation, but the distance between the catheter tip and the target tissue necessary to achieve lethal temperature (indicative of the estimated lesion depth) was less for 90 W/4 s‐vHPSD ablation than for 50 W‐HPSD ablation. Third, both 90 W/4 s‐vHPSD ablation and 50 W‐HPSD ablation were highly effective in generating temperatures needed for transmural lesions. The incidences of phrenic nerve and esophageal adventitia injuries were similar between ablation strategies, but the maximum depth of the injury site from esophageal adventitia was significantly shorter with 90 W/4 s‐vHPSD ablation than with 50 W‐HPSD ablation.

### 4.1 Tissue temperatures in the veno‐atrial junction with 90 W/4 s‐vHPSD and AI‐guided 50 W‐HPSD ablation

This study disclosed actual in vivo time‐versus‐tissue temperature profiles during 90 W/4 s‐vHPSD and AI‐guided 50 W‐HPSD ablation, with tissue temperature having been recorded by thermocouples implanted epicardially at the veno‐atrial junction. Expectedly, 90 W/4 s‐vHPSD produced a more rapid increase to lethal tissue temperatures (≥50°C) than that seen with AI‐guided 50 W HPSD ablation (4.3 ± 2.7 vs. 8.6 ± 3.2 s, respectively; *p* < .001), but maximum epicardial surface tissue temperature was comparably only 49.2 ± 8.4°C versus 50.0 ± 12.1°C (*p* = .69) during 90 W/4 s‐vHPSD and 50 W‐HPSD ablation performed under the temperature‐control mode with the QDOT MICRO catheter. The more rapid increases in tissue temperature with a significantly shorter t‐Tbase (29.9 ± 16.3 vs. 49.5 ± 23.2 s) by 90 W/4 s‐vHPSD ablation versus 50 W‐HPSD ablation may, in part, reflect a greater effect of resistive heating rather than conductive heating on lesion formation by 90 W/4 s‐vHPSD ablation.^7^, ^14^ There have been only a few reports of in vivo or ex vivo tissue temperatures reached with irrigated‐tip RF ablation.^7^, ^14^ In our previous work with the STSF catheter,^7^ tissue temperatures rose rapidly during AI‐guided 50 W‐HSPD ablation (t‐T ≥ 50°C: 7.6 ± 3.6 s), but Tmax was significantly higher (63.8 ± 11.7°C, *p* < .001 vs. that in this study). Unlike the QDOT MICRO catheter, the STSF catheter does not have a temperature‐control mode (the single electrode of the STSF catheter does not adequately reflect temperature of adjacent tissue), so our data suggest potential safety of 50W‐HPSD ablation performed with the QDOT MICRO catheter to prevent excessively high tissue temperatures. In another important study,^14^ Nakagawa et al. investigated tissue temperatures reached in canine thigh muscle during 90 W/4 s‐vHPSD ablation (QDOT MICRO catheter) and during 50 W/10 s‐HPSD ablation (STSF catheter). Tmax at 3‐mm depth (electrode temperature limited to 60°C for the 90 W power setting) was much higher (perpendicular electrode orientation: 85.3 ± 6.7°C; parallel electrode orientation: 83.4 ± 8.8°C vs. 49.2 ± 8.4°C) than that in our model. This may be due to the different tissue models used (canine thigh muscle vs. porcine beating heart), electrode temperature limit (60°C vs. 55°C), and temperature recording sites (inside the tissues vs. epicardial surface), and thus, our model may represent in vivo epicardial surface temperatures at the veno‐atrial junction. Importantly, we showed that the best tip‐to‐tissue distance cut‐point for prediction of lethal temperature (≥50°C) (indicative of the estimated lesion depth) was somewhat shorter 90 W/4 s‐vHPSD ablation than with 50 W‐HPSD ablation (3.13 vs. 4.27 mm). Because of the thin atrial walls, we could not assess actual maximum lesion width and volume, but lesions on the endocardium were significantly wider following 90 W/4 s‐vHPSD ablation than following 50 W‐HPSD ablation (6.8 ± 1.7 vs. 5.0 ± 2.0 mm, *p* = .007). In the first in vivo study of 90 W/4 s‐vHPSD ablation,^5^ 90 W/4 s‐vHPSD ablation (electrode temperature limited to 65°C) with various CF settings was shown to produce wide and shallow lesions (width and depth of 10.36 ± 1.2 mm and 3.62 ± 0.6 mm, respectively in a swine thigh muscle preparation, and 7.0 ± 1.1 mm and 3.39 ± 0.8 mm, respectively, in the atrial wall of beating swine heart). Nakagawa et al.^13^ also showed that 90 W/4 s‐vHPSD ablation (CF of 10 g) with the QDOT MICRO catheter produced slightly wider and shallower lesions than 50 W/10 s‐HPSD (CF of 10 g) with the STSF catheter (parallel orientation: width 8.9 ± 0.5 mm vs. 8.3 ± 0.6 mm and depth 3.9 ± 0.5 vs. 4.9 ± 0.3 mm, respectively, in a thigh muscle preparation, and width 7.2 ± 0.9 vs. 6.7 ± 1.3 mm and depth 3.6 ± 0.6 vs. 4.8 ± 0.9 mm, respectively in the ventricular walls of the beating heart). Takigawa et al. reported similar lesion widths and depths with 90 W/4 s‐vHPSD ablation.^15^ Thus, there is growing evidence that the shallower and wider lesions are created with 90 W/4 s‐vHPSD ablation through a greater effect of resistive heating than of conductive heating than is seen with 50 W‐HPSD ablation.^5^, ^14^, ^15^ Several possible mechanisms are considered in terms of different lesion geometrics between those 2 ablation settings. Several experimental studies on electroporation (using ultrahigh‐voltage and ultra‐short‐duration pulses) have shown a greater electric field and temperature distribution when the electrodes are positioned parallel to the muscle fiber than when positioned perpendicular to the muscle fiber.^16^, ^17^ We did not incorporate catheter orientation into our in vivo assessment, but most target sites were not perpendicular to the catheter tip, explaining, at least in part, why shallow and relatively wide lesions were created by 90 W/4 s‐vHPSD ablation. Contrary to 90 W/4 s‐vHPSD ablation, 50 W‐HPSD ablation can create deeper lesions with a longer RF time, but it might have a greater cooling effect from the irrigation flow, which may hinder the creation of endocardial lesions. Importantly, both 90 W/4 s‐vHPSD ablation and 50 W‐HPSD ablation provided full‐thickness lesions of 0.75−1.93 mm in the SVC, RPV, and IVC walls. Therefore, 90 W/4 s‐vHPSD ablation has been reported to be suitable for thin‐walled structures such as the human atrium, with the walls of the PV in humans being very thin, measuring 0.82–1.24 mm on computed tomography^18^, ^19^, ^20^ and 1.7–2.8 mm on autopsy.^21^ Nonetheless, a recent study of Bortone et al. showed a significantly lower rate of a first‐pass PVI with 90 W/4 s‐vHPSD ablation than 50 W HSPD ablation (37/75 [49.3%] vs. 61/75 [81.3%], *p* < .001).^22^ The failure sites of the first‐pass PVI were mostly the PV carina regions, which are known as thickened veno‐atrial junction sites.^18^, ^19^, ^20^ Therefore, shallower lesions with the 90 W/4 s‐vHPSD ablation implicated that this ablation setting might not be sufficient for thick atrial walls.

### 4.2 Effect of 90 W/4 s‐vHPSD ablation versus AI‐guided 50 W‐HPSD ablation on the adjacent tissues

Our study showed collateral damage with respect to actual temperatures of adjacent tissues, such as the phrenic nerve and esophagus, reached by performance of 90 W/4 s‐vHPSD ablation and 50 W‐HPSD ablation targeting the SVC orifice and the IVC tissues in pigs and mimicking the human inferior PV at close distance to the esophagus. The shallower lesions produced by 90 W/4 s‐vHPSD ablation than by 50 W‐HPSD ablation may potentially reduce collateral damage, but this study showed the incidences of persistent phrenic nerve injury and external esophageal injury to be similar between 90 W/4 s‐vHPSD ablation and 50 W‐HPSD ablation. The average distance from the tip‐to‐thermocouple distance at the phrenic nerve and esophagus was 3.0–4.0 mm, and, in achieving lethal tissue temperature, the tip‐to‐thermocouple distance was 3.13 mm for 90 W/4s‐vHPSD ablation and 4.27 mm for 50 W‐HPSD ablation; thus, we speculate that lethal tissue temperatures of 50°C or more can be reached by both 90 W/4 s‐vHPSD ablation and 50 W‐HPSD ablation. Recent in vivo studies^5^, ^22^ have indicated a potential for collateral damage, such as lung damage, and a clinical study^8^ has indicated a potential for esophageal ulcer hemorrhage, even with 90 W/4 s‐vHPSD ablation. Importantly, we found that esophageal adventitia injury was slightly shallower with 90 W/4 s‐vHSPD ablation than with 50 W‐HPSD ablation. This can be explained by the shorter tip‐to tissue distance required to achieve a lethal temperature of ≥50°C and the lower temperatures of tissues located close to esophagus produced by 90 W/4 s‐vHSPD ablation (50.1 ± 9.7°C vs. 52.6 ± 16.5°C by 50 W‐HPSD).

### 4.3 Clinical implications

Our study of 90 W/4 s‐vHPSD ablation in comparison to 50 W‐HPSD ablation revealed that the shorter tip‐to‐tissue distance (3.13 vs. 4.27 mm) associated with 90 W/4 s‐vHPSD ablation predicts achievement of lethal tissue temperature (≥50°C), and that the 90 W/4 s‐vHPSD ablation produces full‐thickness and significantly wider endocardial atrial lesions. Although 90 W/4 s‐vHPSD ablation theoretically provides transmural lesions even in thin human PV walls, clinical studies of AF ablation showed that first‐pass PVI was achieved in 41 of 52 (78.8%) patients under 90 W/4 s‐vHPSD mode,^5^ and the rate of achievement was even lower with 90 W/4 s‐vHPSD ablation in only 37 of 75 (49.3%) patients.^22^ A possible explanation for the relatively high incidence of failure of a first‐pass PVI was mainly due to thickened veno‐atrial junction sites as indicated above.^14^, ^22^ Those findings together with our results, suggest that a 50 W‐HPSD ablation in the thickened atrial regions in combination with 90 W‐vHPSD ablation in the other regions might be a better strategy that can be used to increase the success rate of a first‐pass PVI. Another important point was that, despite similar tip‐to tissue distances, physiological phrenic nerve injury occurred more quickly with 90 W/4 s‐vHPSD ablation than with 50W‐HPSD ablation (3.2 ± 0.7 vs. 8.5 ± 0.5 s; *p* < .001). t‐T ≥ 50°C was 4.3 ± 2.7 s for 90 W/4 s‐vHPSD and 8.6 ± 3.2 for 50W‐HPSD ablation, respectively. Results of a clinical study in which the STSF was used suggested clinical acceptability of use of 50 W power and ≤4‐s energy delivery to the SVC close to the phrenic nerve.^23^ Therefore, 90 W/4 s‐vHPSD ablation should not be used, rather 50 W‐HPSD with ≤4‐s energy delivery may be a potential option to prevent phrenic nerve injury when SVC isolation is performed. Our most important finding was that despite a similar incidence of esophageal injury, esophageal adventitia injury was shallower with 90 W‐vHPSD than with 50 W‐HPSD ablation. This suggests that esophageal injury caused by 90 W/4 s‐vHPSD ablation might be smaller and more superficial, and thus, preferable than that caused by AI (target value: 400)‐guided 50 W‐HPSD ablation. Or t‐T ≥ 50°C was 8.6 ± 3.2 s during AI‐guided 50 W‐HPSD, and therefore, 50 W‐HPSD ablation with energy delivery lasting ≤5 s may be another option to prevent esophageal injury. Even if 90 W/4 s‐vHPSD ablation or AI‐guided 50 W‐HPSD ablation with energy delivery lasting ≤5 s is used, some distance between lesions (perhaps around 6 mm vs. 4 mm, judging from our endocardial lesion width) and a waiting time (>29.9 vs. >49.5 s based on the t‐Tbase) after a previous energy delivery may minimize esophageal injury during posterior LA wall ablation.

### 4.4 Limitations

Our investigation was limited first by the fact that it was conducted as an in vivo swine study. The thoracic venous tissues and anatomical locations or courses of structures such as the phrenic neve and esophagus in pigs may differ from those in humans. Therefore, we created a model in which we positioned the esophagus close to the IVC to simulate the human anatomy as much as possible. Even in this model, differences in our measurements between the pigs in the in vivo environment should be considered, that is, the tissue temperatures of transmural atrial lesions might not always reflect the center of the lesions, or epicardial adipose tissue may hinder the recording of the actual atrial tissue temperatures. Second, we applied a particular ablation catheter, and thus our data may or may not reflect tissue heating achieved with other systems. Third, we performed ablation only of the thin veno‐atrial junction, so, we could not measure the maximum lesion width, depth, and volume. Therefore, we used the endocardial lesion width and tip‐tissue distance ROC cutoff point for a lethal temperature as alternatives to the lesion width and depth. Finally, the study was designed to investigate distribution of tissue temperatures resulting from 90 W/4 s‐vHPSD ablation (electrode temperature limit: 55°C) as a counterpart to 50 W (electrode temperature limit: 47°C) at a fixed AI value of 400. This point should be taken into account in interpreting our study results. For example, several previous studies reported that a 50 W‐HPSD ablation with the AI set below 400 on the LA posterior or a 50 W‐HPSD with a ≤ 4‐s energy delivery to the SVC might decrease the risk of esophageal injury^24^, ^25^ or phrenic nerve injury.^23^ Further studies will be needed to clarify those points.

## 5 CONCLUSIONS

Actual tissue temperatures reached by 90 W/4 s‐vHPSD ablation targeting the RPV, SVC, and IVC and those recorded at adjacent tissue, that is, the esophagus and phrenic nerve, were shown in our in vivo model to reach ≥50°C more quickly than those reached by AI‐guided 50 W‐HPSD ablation, but the tip‐to‐target tissue distance needed to achieve a lethal temperature of ≥50°C was shorter for 90 W/4 s‐vHPSD ablation. Therefore, there is a possibility that 90 W/4 s‐vHPSD ablation cannot create transmural lesions especially in thick human atrial wall areas such as along the Coumadin ridge. Nevertheless, 90 W/4 s‐vHPSD ablation allows for transmural lesions in our model that are wider than those produced by AI‐guided 50 W‐HPSD ablation. Our data may provide clinical insight into understanding how to achieve complete PV isolation and to prevent collateral damage during 90 W/4 s‐vHPSD ablation and AI‐guided 50 W‐HPSD ablation.

## Data Availability

The data that support the findings of this study are available on request from the corresponding author. The data are not publicly available due to privacy or ethical restrictions.
